# Glucagon-like peptide-1 (GLP-1) levels are associated with acute kidney injury after cardiac surgery

**DOI:** 10.1038/s41598-026-48483-6

**Published:** 2026-04-25

**Authors:** Agnieszka Szafran, Berkan Kurt, Jessica Pracht, Elisabeth Zechendorf, Thomas Breuer, Christian Stoppe, Gernot Marx, Frank Tacke, Florian Kahles, Jana C Mossanen

**Affiliations:** 1 Department of Intensive and Intermediate Care, University Hospital, RWTH Aachen, Aachen, Germany; 2 Department of Internal Medicine I - Cardiology, University Hospital RWTH Aachen, Aachen, Germany; 3 Department of Anaesthesiology, Intensive Care, Emergency and Pain Medicine, University Hospital Würzburg, Würzburg, Germany; 4 Department of Cardiac Anesthesiology & Intensive Care Medicine, Charité Berlin, Berlin, Germany; 5 DZHK (German Center for Cardiovascular Research), partner site Berlin, Berlin, Berlin, Germany; 6 Department of Hepatology and Gastroenterology, Campus Virchow-Klinikum and campus Charité – Universitätsmedizin Berlin, Berlin, Germany; 7 Department of Anaesthesiology, University Hospital RWTH Aachen, Pauwelsstr. 30, D- 52074 Aachen, Germany

**Keywords:** GLP-1, GIP, Acute kidney injury, Cardiac surgery, Biomarkers, Incretin hormones, Perioperative medicine, Critical care, Biomarkers, Cardiology, Diseases, Medical research, Nephrology

## Abstract

Acute kidney injury (AKI) is a frequent and clinically relevant complication after cardiac surgery. Early prediction remains challenging because serum creatinine is a delayed and insensitive marker of renal dysfunction. Glucagon-like peptide-1 (GLP-1) and glucose-dependent insulinotropic polypeptide (GIP) are incretin hormones with established metabolic functions and emerging roles in cardiovascular, renal, and critical illness–related pathophysiology, and their perioperative association with AKI remains incompletely characterised. In this prospective single-centre observational study, 107 adults undergoing elective cardiac surgery were enrolled. Serum GLP-1 and GIP were measured at four time points (preoperatively, immediately postoperatively, and on postoperative days 1 and 4). AKI was defined according to KDIGO criteria. Subgroup analyses excluded patients with chronic kidney disease (CKD). Postoperative AKI occurred in 21 patients (19.6%), predominantly KDIGO stage 1. These patients had longer ICU stays (5 [2–12] vs. 3 [1–10] days; *p* < 0.001), higher SAPS II scores (35 [28–39] vs. 29 [25–33]; *p* = 0.027), and higher SOFA scores on postoperative days 1 and 4 (both *p* < 0.05). Preoperative GLP-1 levels were significantly elevated in patients who developed AKI (38.3 [30–52] vs. 27.4 [16.7–48.9] pmol/L; *p* = 0.04), whereas baseline creatinine and GIP did not differ. The association between GLP-1 and AKI remained significant after excluding patients with CKD. Preoperative endogenous GLP-1 levels were associated with postoperative AKI and were elevated earlier in the perioperative course compared with serum creatinine, particularly in patients without CKD. These findings suggest that GLP-1 may reflect early renal stress or vulnerability and warrant further investigation in larger cohorts.

**Supplementary Information:**

The online version contains supplementary material available at 10.1038/s41598-026-48483-6.

## Introduction

GLP-1 and GIP have emerged as key regulatory peptides with growing relevance in cardiometabolic and renal medicine. Initially studied for their glucose-lowering and insulinotropic properties, GLP-1 receptor agonists (GLP-1RA) and dual GIP/GLP-1 RAs have gained clinical interest for the treatment of type 2 diabetes and obesity. Beyond glycaemic control, GLP-1RAs have demonstrated consistent cardiovascular and renal benefits, including reductions in body weight, blood pressure, and major adverse cardiovascular events (MACE)^1,2^. These effects have been observed across large cardiovascular outcome trials and confirmed in meta-analyses showing reductions in cardiovascular and all-cause mortality, heart failure hospitalisations and composite renal endpoints^3^. Evidence for renoprotective effects first emerged from cardiovascular outcome trials reporting attenuation of albuminuria progression and slower eGFR decline in patients treated with GLP‑1RAs^2,4^. These findings were recently substantiated in the FLOW trial, which demonstrated significant renal benefits of semaglutide across kidney failure, sustained decline in estimated glomerular filtration rate (eGFR) and kidney- or cardiovascular-related mortality in patients with type 2 diabetes and CKD^5^. Subsequent meta-analyses further confirmed consistent improvements in composite kidney outcomes across diverse populations^3,6^. The SELECT trial extended these observations to non-diabetic populations, reporting renal benefits of semaglutide in patients without diabetes or CKD^4,6^. These data suggest that GLP‑1RAs may be particularly beneficial for patients with CKD. In addition to their metabolic effects, GLP-1RAs have been associated with broader physiological changes, including effects on fluid and electrolyte homeostasis^7^. Parallel to therapeutic data, observational studies in non-surgical settings—including acute heart failure and critical illness—have linked elevated endogenous GLP‑1 levels with renal dysfunction and systemic inflammation, suggesting a potential role in AKI pathophysiology^6^. However, the relevance of endogenous GLP‑1 as an early biomarker of renal vulnerability in the perioperative setting remains poorly understood.

AKI is a frequent and serious complication after cardiac surgery, with an incidence of up to 30% depending on patient risk and procedure type^8,9^. Even mild AKI is independently associated with increased short- and long-term mortality^10^. Diagnosis remains based on serum creatinine, a delayed and insensitive marker of acute renal dysfunction^11,12^. Several biomarkers such as neutrophil gelatinase‑associated lipocalin (NGAL), interleukin‑18 (IL‑18) and soluble urokinase-type plasminogen activator receptor (suPAR) have been evaluated^13^, yet most reflect manifest renal injury rather than early functional stress, limiting their ability to guide timely preventive strategies. In this context, we extended our biomarker analysis to the incretin axis and investigated the association between perioperative GLP-1 and GIP serum levels and the occurrence of postoperative AKI.

## Methods

### Study design and patient characteristics

This prospective, single-centre observational study was conducted at the Department of Intensive and Intermediate Care, University Hospital RWTH Aachen, Germany. The present analysis represents a secondary, hypothesis-generating post-hoc analysis of an ongoing prospective biobank study (ClinicalTrials.gov identifier: NCT02488876)^13^. While the parent study aims to enroll approximately 1000 patients undergoing cardiac surgery, the current analysis was conducted on a subset of 107 consecutively enrolled adult patients (≥ 18 years) undergoing elective cardiac surgery with cardiopulmonary bypass, for whom complete biomarker data (GLP-1 and GIP) and postoperative AKI outcomes were available at the time of analysis. Recruitment into the parent biobank is ongoing and limited primarily by logistical and laboratory resources; no additional selection criteria beyond sample availability were applied.

The study was approved by the institutional ethics committee (Approval No. 151/09) and conducted in accordance with the Declaration of Helsinki. Written informed consent was obtained from all participants. Exclusion criteria included emergency surgery, severe hepatic impairment (Child-Pugh C), active infection, or refusal to participate.

### Clinical data and outcome definitions

Patient data, clinical information and laboratory measurements were prospectively collected at four predefined time points: preoperatively, immediately postoperatively, and on postoperative days 1 and 4. Acute kidney injury (AKI) was defined according to the Kidney Disease: Improving Global Outcomes (KDIGO) criteria as an increase in serum creatinine ≥ 0.3 mg/dL within 48 h or ≥ 1.5-fold from baseline, and staged (KDIGO stages 1–3) according to the maximum creatinine increase. Pre‑existing CKD was defined as eGFR < 60 mL/min/1.73 m² for > 3 months before surgery; two patients with CKD stage V were on chronic haemodialysis. Patients were screened daily for AKI during the first four postoperative days^14^. Follow‑up information was obtained through direct contact with patients, relatives, or primary physicians. None of the patients received GLP-1 receptor agonists or dipeptidyl peptidase-4 (DPP-4) inhibitors. A small proportion of patients were treated with sodium–glucose cotransporter-2 (SGLT-2) inhibitors at baseline. As SGLT-2 inhibitors do not directly affect GLP-1 levels, the measured GLP-1 concentrations were considered to reflect circulating endogenous GLP-1 levels.

### Determination of serum concentrations of GIP, GLP-1 and creatinine

Preoperative serum samples were available for all 107 patients; postoperative samples for 93 patients; day‑1 samples for 95 patients; and day‑4 samples for 70 patients. Missing samples at later time points were primarily related to early ICU discharge and clinical workflow factors. All samples were immediately cooled on ice and stored at − 80 °C after centrifugation. GIP and GLP‑1 concentrations were measured using commercial enzyme-linked immunosorbent assay (ELISA) kits (GIP: Human GIP (Total), Catnr. EZHGIP-54 K, Millipore, Range: 4.2 pg/ml to 2000pg/ml; GLP-1: GLP-1 Total, Cat. No. EZGLP1T-36 K, Millipore, Range: 4.1 pM to 1000 pM). Creatinine was measured using routine clinical methods. Laboratory personnel were blinded to clinical data and AKI status.

### Management of anesthesia and surgical procedures

General anesthesia followed institutional standards as previously described^13^. Intraoperative fluid management consisted of crystalloid infusion at 1 mL·kg⁻¹·h⁻¹. All patients were transferred to the intensive care unit (ICU) postoperatively and treated according to local protocols. Conventional cardiopulmonary bypass (CPB, “on-pump”) was performed using non‑pulsatile flow (2.2 L·min⁻¹·m²) with a mean arterial pressure target of 50–70 mmHg. Cardiac arrest was induced by a single antegrade infusion of cold crystalloid cardioplegia. Heparin was antagonized with protamine at a 1:1 ratio after CPB. Aspirin was given 8 h postoperatively. Off‑pump procedures followed established institutional techniques as previously described^15^.

Surgical procedures comprised isolated CABG (*n* = 54, 51%), combined CABG with aortic valve replacement (*n* = 17, 16%), isolated aortic valve replacement (*n* = 9, 8%), complex aortic root surgery including Bentall, David or Hemashield procedures (*n* = 8, 7%), and other interventions (*n* = 28, 26%).

### Statistical analysis

Continuous variables were tested for normality using the Shapiro–Wilk test. As most variables were not normally distributed, data are presented as median and interquartile range (IQR). Differences between groups were analyzed using the Mann–Whitney U test; multiple‑group comparisons used Kruskal–Wallis ANOVA with post hoc Mann–Whitney testing. Spearman’s rank correlation assessed associations between continuous variables.

Receiver–operating characteristic (ROC) analysis was used to evaluate predictive performance of GLP‑1 for postoperative AKI. Optimal cut‑off values were derived using the Youden Index, with calculation of sensitivity, specificity, likelihood ratios, and diagnostic odds ratios. Boxplots display all data including outliers. Additional analyses were performed to further assess the association between preoperative GLP-1 levels and postoperative AKI. Univariable and multivariable logistic regression analyses were conducted using a dichotomized GLP-1 variable based on the ROC-derived Youden cut-off. Given the limited number of AKI events, separate multivariable models were constructed to minimize the risk of overfitting. Time-to-event analyses were performed using Kaplan–Meier estimates and Cox proportional hazards regression. Differences between groups were assessed using the log-rank test. Cox regression analyses were performed in a univariable and exploratory multivariable manner adjusting for selected clinical variables. A p‑value < 0.05 was considered statistically significant.

All statistical analyses were performed using SPSS version 29 (IBM SPSS Statistics, Chicago, IL, USA)^13^.

## Results

### Patient characteristics and incidence of acute kidney injury

Clinical characteristics of 107 patients included in this prospective study are summarized in Table 1. Two patients were excluded due to incomplete postoperative laboratory data, resulting in 105 complete datasets for final analysis. According to the collective of cardiac surgery patients, the majority of patients presented cardiovascular comorbidities and received appropriate guideline-directed medical therapy.

**Table 1:** Patient characteristics.

Parameter	All patients	Non-AKI	AKI	*p*-value
	*n* = 107	*n* = 86	*n* = 21	
**Demographics**				
Sex (male/female)	77/30	62/24	15/6	0.952
Age median (IQR) (years)	69 (61–76)	67 (61–75)	75 (66–79)	0.062
Body mass index (IQR) (BMI)	27 (24–30)	27 (24–30)	27 (25–30)	0.613
30 days Mortality *n* (%)	1 (1)	0 (0)	1 (5)	0.186
90 days Mortality *n* (%)	3 (3)	1 (1)	2 (10)	0.110
**Surgery and ICU observation**				
CABG *n* (%)	54 (51)	47 (55)	7 (32)	0.080
CABG + AVR *n* (%)	17 (16)	10 (12)	7 (32)	**0.015**
AVR *n* (%)	9 (8)	6 (7)	3 (14)	0.279
Bentall, David or Hemashield *n* (%)	8 (7)	7 (8)	1 (5)	0.598
Other cardiac surgery *n* (%)	28 (26)	16 (19)	3 (13)	0.642
Ischemia time (IQR) (min)	79 (60–110)	79 (60–109)	83 (60–114)	0.750
Time of CBP (IQR) (min)	125 (101–156)	125 (100–160)	124 (101–156)	0.729
Total time of surgery (min)	251 (220–290)	252 (217–287)	249 (220–315)	0.476
**Postoperative period**				
ICU days median (IQR) (days)	3 (1–12)	3 (1–10)	5 (2–12)	**< 0.001**
SAPS day1 median (IQR)	29 (26–35)	29 (25–33)	35 (28–39)	0.027
SAPS day4 median (IQR)	24 (19–31)	22 (17–28)	29 (21–37)	0.060
SOFA day1 median (IQR)	5 (3–7)	5 (3–6)	6 (5–7)	0.011
SOFA day4 median (IQR)	0 (0–3)	0 (0–2)	3 (1–5)	**< 0.001**
Nephrotoxic antibiotics *n* (%)	3 (3)	2 (2)	1 (5)	0.484
Dialysis *n* (%)	3 (3)	2 (2)	1 (5)	0.484
**AKI stages**				
Stage I *n* (%)	17 (16)	0 (0)	17 (81)	
Stage II *n* (%)	3 (3)	0 (0)	3 (14)	
Stage III *n* (%)	1 (1)	0 (0)	1 (5)	
**Comorbidities**				
Type 2 diabetes *n* (%)	39 (36)	32 (37)	7 (33)	0.741
Hypertension *n* (%)	81 (76)	64 (74)	17 (81)	0.531
Chronic kidney disease *n* (%)	10 (9)	5 (6)	5 (23)	**0.011**
**Medication**				
Diuretics use *n* (%)	54 (50)	37 (43)	17 (81)	**0.002**
β–blocker use *n* (%)	75 (70)	61 (71)	14 (67)	0.702
AT II receptor antagonist use *n* (%)	24 (22)	16 (19)	8 (36)	0.055
ACE inhibitors use *n* (%)	52 (49)	43 (50)	9 (43)	0.557
Statin use *n* (%)	67 (63)	53 (62)	14 (64)	0.669
Calcium channel blocker use *n* (%)	24 (22)	16 (9)	8 (36)	0.055
SGLT2 inhibitor use n (%)	5 (5)	3 (3)	2 (10)	0.200
**Laboratory parameters pre surgery**				
WBC median (IQR) (×10^3^ µL^− 1^)	7.9 (6.4–10.5)	7.5 (6.2–9.9)	9.0 (8.2–13.3)	**0.009**
Creatinine pre-OP (IQR) (mg·dL^− 1^)	1 (0.74–1.1)	0.9 (0.74–1.1)	1 (0.7–1.1)	0.269
eGFR pre-OP (IQR) (mL·min^− 1^)	75 (63–90)	77 (65–90)	70 (48–90)	0.233
GIP pre-OP (IQR) (pg/mL)	31.1 (19.9–52)	28.9 (18.5–51.6)	33.8 (12.2–60.9)	0.324
GLP-1 pre-OP (IQR) (pmol/L)	30.1 (18.4–49.3)	27.4 (16.7–48.9)	38.3 (30–52)	**0.042**

AKI: Acute kidney injury; ACE: Angiotensin-converting-enzyme; AT: Angiotensin; AVR: Aortic valve repair; CABG: Coronary artery bypass graft; CBP: Cardiopulmonary bypass; eGFR: Estimated glomerual filtration rate; ICU: Intensive care unit; IQR: Interquartile range; OP: Operation; GIP: Glucose-dependent insulinotropic polypeptide; SAPS: Simplified acute physiology score; SOFA: Sequential organ failure assessment; Nephrotoxic antibiotics included aminoglycosides (e.g., gentamicin), vancomycin, and piperacillin-tazobactam; SGLT2: Sodium-glucose co-transporter 2; GIP: glucose-dependent insulinotropic polypeptide; GLP1: glucagon-like peptide-1; WBC: White blood cell count.

For comparing continuous variables, the Mann-Whitney U Test was performed. For comparing dichotomous variables, the Chi2-Test was performed.

Postoperative AKI occurred in 21 patients (19.6%), with a median onset on postoperative day 1, occurring within the first four days after surgery. The distribution of AKI onset was as follows: 4 cases occurred on the day of surgery, 8 on postoperative day 1, 3 on day 2, 3 on day 3, and 3 on day 4. Among patients without pre-existing CKD (*n* = 97), postoperative AKI occurred in 16 patients (16.6%). Most AKI cases were classified as stage 1 (17 patients, 81.0%), followed by stage 2 (3 patients, 14.3%) and stage 3 (1 patient, 4.7%). Patients who developed AKI had a higher prevalence of pre-existing CKD, more frequent use of diuretics, and higher baseline white blood cell counts. In addition, these patients were more likely to undergo combined surgical procedures, including double CABG and valve replacement. Despite these more extensive procedures, total operative times did not differ significantly between patients with and without postoperative AKI.

Patients who developed AKI exhibited significantly higher Simplified Acute Physiology Score (SAPS) on postoperative day 1 and elevated Sequential Organ Failure Assessment (SOFA) scores on day 1 and day 4. These patients also required longer stays in the intensive care unit (ICU) compared to patients without AKI (median 5 [2–12] days vs. 3 [1–10] days; *p* < 0.001). No significant differences were observed in myocardial ischemia time or use of angiotensin-converting enzyme (ACE) inhibitors, angiotensin II receptor blockers (ARBs), statins, or calcium channel blockers. Notably, none of the included patients received GLP‑1 receptor agonists or dipeptidyl peptidase-4 (DPP-4) inhibitors preoperatively.

Group comparisons demonstrated significant differences between AKI and pre‑existing CKD (*p* = 0.011), elevated leukocyte count (*p* = 0.009), and diuretic use (*p* = 0.002), whereas age, duration of surgery, and CPB time were not associated with AKI (Table 1).

### Preoperative GLP-1, but not GIP or creatinine, is elevated in patients developing postoperative AKI

**Fig. 1:**
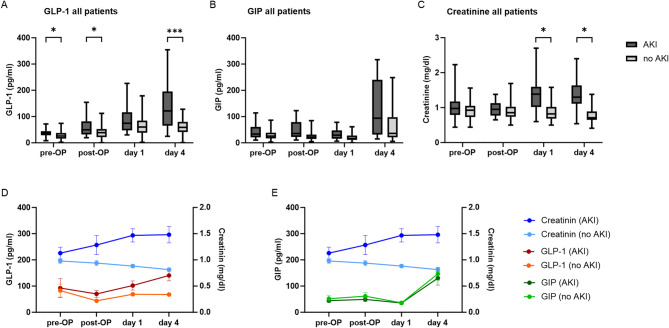
Serum levels of GLP-1, GIP, and creatinine in patients with and without postoperative acute kidney injury (AKI). Box-and-whisker plots of median serum GLP-1 levels in patients with and without postoperative AKI at four time points. **(A)** GLP-1 levels were significantly higher in the AKI group preoperatively (*p* = 0.042), postoperatively (*p* = 0.029) and on postoperative day 4 (*p* < 0.001). **(B)** GIP serum levels show no significant differences. **(C)** Creatinine was elevated in patients with AKI on postoperative days. **(D)** Mean serum concentrations of GLP-1 and serum creatinine in patients with and without postoperative AKI. **(E)** Mean serum concentrations of GIP and creatinine in the same patient groups. Outliers were excluded from the graphical presentation but included in all statistical analyses. Box plots are displayed where the bold line indicates the median per group and horizontal lines show minimum and maximum values of calculated non-outlier values (* *p* < 0.05, ** *p* < 0.01, *** *p* < 0.001).

Serum levels of GLP-1, GIP, and creatinine were measured at four time points: preoperatively, immediately postoperatively, and on postoperative days 1 and 4. In patients who developed postoperative AKI, preoperative GLP-1 levels were significantly higher compared to those without AKI (median 38.3 [30–52] pmol/L vs. 27.4 [16.7–48.9]; *p* = 0.042; Fig. 1A) and continued to rise postoperatively, with the most pronounced difference observed on day 4 (*p* < 0.001).

In contrast, no significant differences in GIP concentrations (all *p* > 0.10) were observed between groups, although a slight, delayed increase was seen on postoperative day 4 without reaching statistical significance (*p* = 0.40; Fig. 1B). Serum creatinine levels increased postoperatively in AKI patients but showed considerable overlap with the non-AKI group at all time points (Fig. 1C).

While preoperative GLP-1 levels were significantly higher in patients developing postoperative AKI, serum creatinine levels diverged only after surgery, with significant differences observed from postoperative day 1 onwards (Fig. 1D).

GIP levels rose in both groups over time, particularly on postoperative day 4, but no significant intergroup differences were observed (Fig. 1E).

### Preoperative GLP-1, but not GIP or creatinine, is elevated in patients without pre-existing CKD

**Fig. 2:**
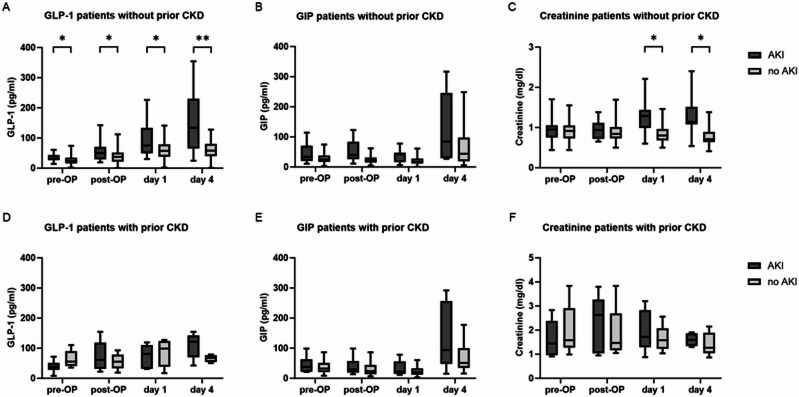
Perioperative serum levels of GLP-1, GIP, and creatinine in patients without pre-existing CKD and with pre-existing CKD. **(A-C)** Box-and-whisker plots of serum GLP-1 levels at four time points in patients without prior CKD. **(A)** GLP-1 levels were significantly higher in patients who developed AKI both preoperatively and on postoperative days 1 and 4 (*p < 0.05; ***p < 0.001). **(B)** Serum GIP levels across the same perioperative time points. **(C)** Serum creatinine levels. Significant group differences between AKI and non-AKI patients emerged on days 1 and 4 (*p < 0.05). **(D-F)** Serum GLP-1 in patients with prior CKD (*n* = 10 patients with 5 patients developing AKI post-surgery). **(D)** No significant differences in GLP-1 levels were observed between patients with and without AKI at any time point. **(E)** Serum GIP levels remained similar between groups across the perioperative period. **(F)** Serum creatinine levels over time. Baseline creatinine values were elevated in both groups, and no distinct group separation was observed throughout the observation period. Outliers were excluded from the graphical presentation but included in all statistical analyses. Box plots display median values (horizontal line), interquartile range (box), and minimum/maximum of non-outlier values (whiskers). (* *p* < 0.05, ** *p* < 0.01, *** *p* < 0.001)

In patients without pre-existing CKD, significant differences in GLP-1 levels were observed over time when comparing patients developing AKI with those who did not (Fig. 2A). GLP-1 levels were already elevated preoperatively (*p* < 0.05) and showed a progressive increase postoperatively, with the most pronounced difference observed on day 4 (*p* < 0.001; Fig. 2A).

In contrast, GIP concentrations did not differ significantly between groups at any time point (Fig. 2B).

Creatinine levels increased significantly in AKI patients by postoperative day 1 and remained elevated on day 4 (*p* < 0.0001; Fig. 2C).

### Preoperative GLP-1, GIP and creatinine levels do not distinguish AKI in patients with pre-existing CKD

Although the number of patients with pre-existing CKD was limited, this high-risk subgroup was included due to its clinical relevance (CKD subgroup 10 patients with 5 patients developing AKI post-surgery). GLP‑1 levels were elevated at baseline but showed minimal dynamic variation and no significant differences between AKI and non‑AKI patients (Fig. 2D). GIP values remained low with high variability on day 4 (Fig. 2E). Creatinine values were elevated in both groups throughout and did not reliably distinguish AKI (Fig. 2F). The subgroup of patients with CKD was included primarily for completeness, and no causal or mechanistic conclusions were drawn from these observations.

### Preoperative GLP-1 shows higher discriminatory ability for postoperative AKI

**Fig. 3:**
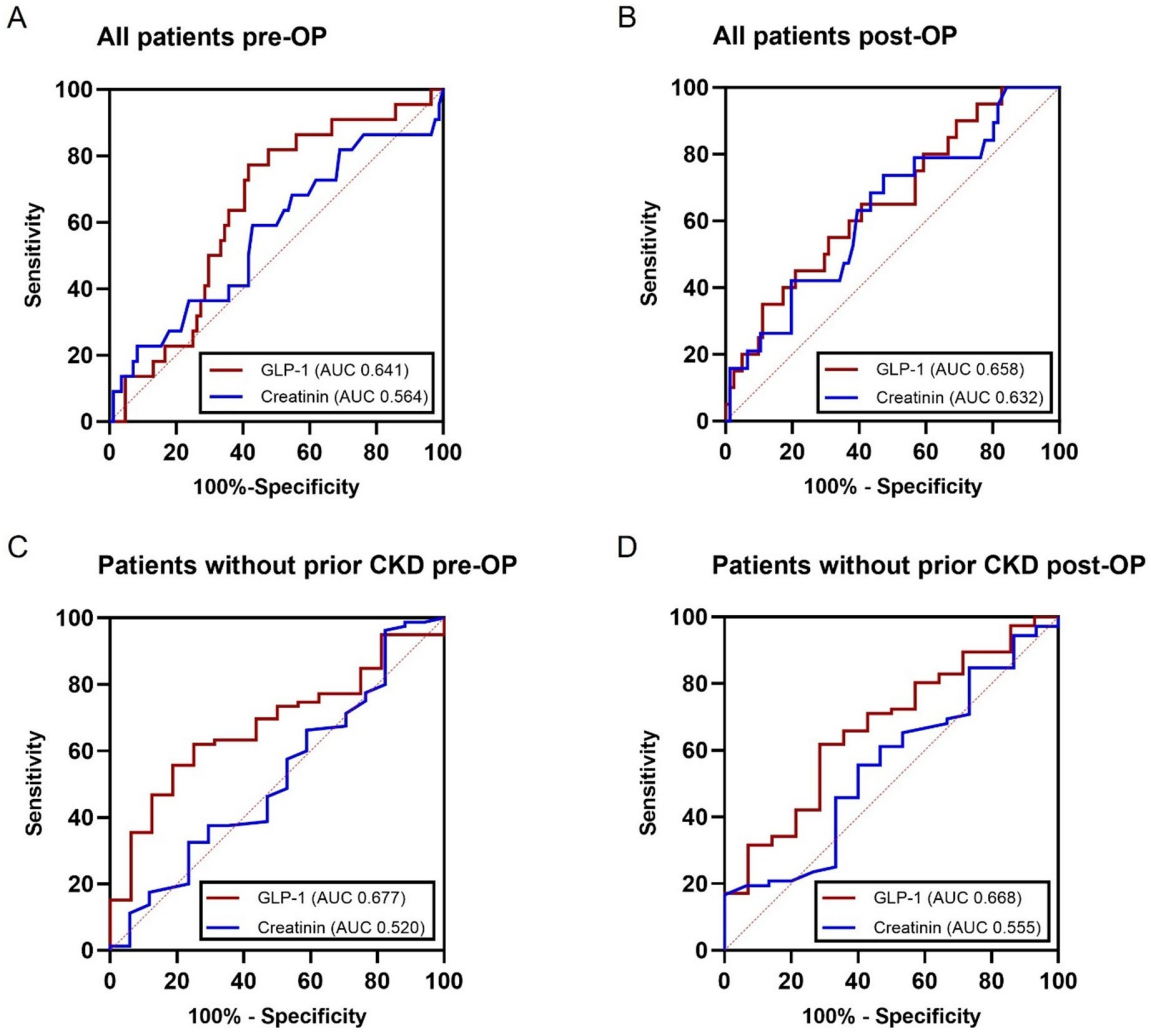
Receiver operating characteristic (ROC) curves comparing the predictive performance of preoperative and postoperative serum GLP-1 and creatinine concentrations for the development of postoperative AKI in all patients and in patients without pre-existing CKD. **(A)** ROC analysis of preoperative GLP-1 (red line; AUC 0.641, 95% CI 0.5210–0.7614) and serum creatinine (blue line; AUC 0.564, 95% CI 0.4220–0.7057) in all patients (*n* = 107). **(B)** ROC analysis of postoperative GLP-1 (AUC 0.658, 95% CI 0.5262–0.7899) and creatinine (AUC 0.632, 95% CI 0.4923–0.7715) measured immediately after surgery. **(C)** ROC analysis of preoperative GLP-1 (red line; AUC 0.677, 95% CI 0.5505–0.8039) and creatinine (blue line; AUC 0.520, 95% CI 0.3638–0.6766) in patients without prior CKD (*n* = 97). **(D)** ROC analysis of postoperative GLP-1 (AUC 0.668, 95% CI 0.5203–0.8162) and creatinine (AUC 0.555, 95% CI 0.3972–0.7121) measured immediately after surgery in the same subgroup.

In ROC analysis of all patients, preoperative GLP-1 levels demonstrated consistently higher AUC values than serum creatinine for the prediction of postoperative AKI (AUC 0.641 vs. 0.564; Fig. 3A). Immediately postoperatively, both markers showed improved discriminatory performance, with GLP-1 maintaining a numerically higher AUC (AUC 0.658 vs. 0.632; Fig. 3B). The corresponding 95% confidence intervals for all AUC values are provided in Fig. 3 indicating moderate precision and overlap between markers.

Both markers exhibited higher postoperative AUCs compared with preoperative measurements, indicating improved discrimination after surgery. GLP-1 remained slightly superior across all time points, suggesting a more consistent and robust predictive capacity throughout the perioperative course. To further characterize the discriminatory performance of GLP-1, optimal cut-off values were determined using the Youden Index (Table 2). In the overall cohort, a preoperative threshold of 30.3 pmol/L yielded a sensitivity of 0.77 and specificity of 0.58 (Youden Index 0.36), while a postoperative threshold of 42.1 pmol/L showed a sensitivity of 0.65 and specificity of 0.59. These values indicate moderate discriminatory accuracy, consistent with the AUC results.

**Table 2:** Diagnostic accuracy of GLP-1 cut-off values for AKI prediction.

	CutOff	Sensitivity	Specificity	Youden Index	Positive LR	Negative LR	Diagnostic Odds Ratio
All pre-OP	30.275	0.7727	0.5833	0.3561	1.855	0.39	4.76
All post-Op	42.095	0.65	0.5926	0.2426	1.595	0.591	2.701
Without prior CKD pre-OP	30.275	0.75	0.6203	0.3703	1.975	0.403	4.9
Without prior CKD post-OP	42.095	0.7143	0.6184	0.3327	1.872	0.462	4.052

### Preoperative GLP-1 shows higher discriminatory ability in patients without prior CKD

To assess whether the association between GLP-1 and AKI was influenced by pre-existing CKD, ROC analyses were repeated in patients without CKD (Fig. 3C and D). In this subgroup, preoperative serum GLP-1 levels showed higher AUCs for predicting postoperative AKI compared with creatinine (AUC 0.677 vs. 0.520; Fig. 3C).

This trend persisted in early postoperative measurements (AUC 0.668 vs. 0.555; Fig. 3D), indicating a consistent trend toward better discrimination with GLP-1, particularly in patients with preserved baseline renal function. Although these differences did not reach statistical significance, GLP-1 showed a reproducible pattern of higher AUCs across all time points, indicating stable discriminatory performance in patients without chronic kidney disease. In patients without CKD, the corresponding Youden-based cut-offs were similar to the overall cohort, with preoperative and postoperative thresholds of 30.3 pmol/L and 42.1 pmol/L, respectively. These cut-offs yielded sensitivities of 0.75 and 0.71, and specificities of 0.62 at both time points, reflecting consistent performance across subgroups.

### Association of preoperative GLP-1 with postoperative AKI

In univariable logistic regression analysis, preoperative GLP-1 levels above the ROC-derived Youden cut-off were significantly associated with postoperative AKI (OR 4.76; 95% CI 1.61–14.12; *p* = 0.005). In exploratory multivariable logistic regression models adjusting for selected clinical variables, including age and sex, BMI and creatinine, baseline renal function and leukocyte count, as well as pre-existing CKD and surgical complexity, this association remained consistent across all models (Table 3). Kaplan–Meier analysis demonstrated a significant separation of AKI-free survival curves between patients with preoperative GLP-1 levels above versus below the Youden cut-off (log-rank *p* = 0.003; supplementary Fig. 4), with lower AKI-free survival in patients with elevated GLP-1 levels. Consistently, in univariable Cox regression, elevated preoperative GLP-1 levels were associated with an increased risk of postoperative AKI (HR 3.95; 95% CI 1.46–10.72; *p* = 0.007). Exploratory multivariable Cox regression analyses yielded directionally consistent results.

**Table 3:** Association of preoperative GLP-1 levels with postoperative AKI: univariable and exploratory multivariable logistic regression analyses.

	*n*	AKI events	OR (95% CI)	*p*
**Univariable**				
GLP-1*	106	22	4.76 (1.61, 14.12)	0.005
**Multivariable**				
Model 1	106	22	5.12 (1.60, 16.40)	0.006
Model 2	105	22	4.71 (1.55, 14.35)	0.006
Model 3	103	22	4.88 (1.58, 15.00)	0.006
Model 4	106	22	6.54 (1.87, 22.84)	0.003

Model 1: Adjusted for age and sex, Model 2: Adjusted for BMI and creatinine, Model 3: Adjusted for eGFR and leucocyte count, Model 4: Adjusted for pre-existing CKD and complexity of surgery, *Preoperative GLP-1 levels above the ROC-derived Youden cut-off (30.3 pmol/L).

Multivariable models were performed in an exploratory manner due to the limited number of events.

## Discussion

In this prospective study, we investigated the association of the incretin hormones GLP-1 and GIP with postoperative AKI in patients undergoing cardiac surgery. To our knowledge, this is the first clinical study to identify perioperative elevations of GLP-1 as a marker associated with subsequent AKI development in patients undergoing elective cardiac surgery. GLP-1 exhibits favorable biochemical properties, including plasma stability and minimal circadian variation, making it well-suited for perioperative monitoring^16^. Our data extend prior observations linking GLP-1 with renal dysfunction in non-surgical settings and suggests that GLP-1 levels were elevated earlier in the perioperative course compared with serum creatinine, particularly in patients without pre-existing CKD. ROC curve analysis supported this hypothesis, showing a moderate but consistent discriminatory ability of preoperative GLP-1 (AUC 0.677) compared with creatinine (AUC 0.520). While absolute discrimination remained modest (< 0.70), the stability of GLP-1 performance across perioperative time points underscores the consistency of this association across perioperative time points. These findings were further supported by regression and time-to-event analyses, which consistently demonstrated an association between elevated preoperative GLP-1 levels and postoperative AKI across different analytical approaches. While these analyses strengthen the observed relationship, they were performed in an exploratory manner and should be interpreted with caution given the limited number of events. The optimal GLP-1 thresholds derived from Youden Index analysis (≈ 30 pmol/L preoperatively and ≈ 42 pmol/L postoperatively) provided only moderate sensitivity and specificity, which is consistent with the observed AUC values. Nevertheless, these cut-offs may help define clinically relevant ranges for further evaluation in independent cohorts.

Time-course analyses revealed that GLP-1 concentrations were already elevated preoperatively in patients who subsequently developed AKI and continued to rise postoperatively. A transient decrease immediately after cardiopulmonary bypass was followed by a progressive postoperative increase (Fig. 1D), likely reflecting perioperative splanchnic hypoperfusion and stress-induced hormonal fluctuations^17^. The most pronounced differences between the AKI and non-AKI groups were observed on postoperative day 4. In contrast, creatinine concentrations showed only delayed and partially overlapping changes, highlighting its role as a diagnostic rather than a prognostic marker. These results suggest that GLP-1 may reflect both acute changes in kidney function and early renal stress or subclinical damage preceding creatinine-based AKI diagnosis. This hypothesis is supported by findings from Lebherz et al., who first demonstrated an association between elevated GLP-1 levels and albuminuria—independent of glycemic status—in patients with type 2 diabetes, suggesting a potential link between GLP-1 and renal impairment^18^. Alternatively, the selective postoperative increase in GLP-1 observed in AKI patients may represent a compensatory or stress-related response to AKI itself, in line with earlier observations in acute cardiovascular and inflammatory settings^19^.

In contrast to GLP-1, GIP showed no meaningful association with AKI at any perioperative time point, suggesting limited diagnostic utility in this setting. These results align with reports of blunted or dysregulated GIP responses in CKD^20^ and the observation that renal impairment alters incretin clearance and pharmacokinetics^21^.

Among patients without CKD, GLP-1 showed higher AUC values, whereas in those with pre-existing renal impairment, baseline GLP-1 levels were already elevated, and dynamic changes were attenuated. This may reflect reduced renal clearance and altered metabolism of GLP-1 in CKD, potentially attenuating its association with AKI. These findings are consistent with recent evidence from large meta-analyses demonstrating beneficial renal and cardiovascular effects of GLP-1RA in patients with and without type 2 diabetes^22^. The greater clinical challenge, however, lies in identifying patients with normal baseline kidney function who are nonetheless at risk of postoperative AKI. Improved biomarkers are therefore required to detect this unrecognized high-risk subgroup^23^. The number of AKI events in the non-CKD subgroup was limited (non-CKD subgroup n = 97,with n = 16 post-surgery AKI events), and results should therefore be interpreted cautiously as exploratory; formal interaction testing was not performed due to limited statistical power. In addition, the heterogeneous surgical procedures may represent a source of confounding. Although patients with AKI more frequently underwent combined procedures, operative duration and cardiopulmonary bypass time did not differ between groups. Procedure-related confounding cannot be fully excluded. The mechanisms underlying elevated GLP-1 levels in AKI remain incompletely understood. Beyond its metabolic actions, GLP-1 exerts pleiotropic cardio- and renoprotective effects, including anti-inflammatory, endothelial-stabilizing, and natriuretic properties^3,24^. Elevated GLP-1 levels in AKI may reflect a protective or injury-related response, potentially driven by both systemic inflammation and reduced renal clearance. Experimental data demonstrate that inflammatory mediators such as IL-1β, IL-6, and endotoxin can stimulate GLP-1 secretion, and that elevated GLP-1 levels in CKD and critical illness correlate with impaired clearance and inflammatory activity^18,25–27^. Elevated GLP-1 concentrations observed in acute myocardial infarction and critical illness support the interpretation of GLP-1 as a stress-responsive hormone involved in systemic injury and inflammation^19^. However, it remains unclear whether elevated GLP-1 reflects renal vulnerability or a nonspecific systemic stress response. Accordingly, our findings should not be interpreted as evidence of a causal role of GLP-1 in AKI, but rather as supporting its potential utility as an early biomarker of systemic stress.

In addition to its potential biomarker role, GLP-1 signaling may represent an area of interest for further mechanistic investigation. Emerging evidence from GLP-1RA trials demonstrates kidney-protective effects in CKD, raising the question whether early endogenous GLP-1 elevations reflect a compensatory, potentially modifiable pathway. Although this remains speculative, our findings provide a clinical rationale for future studies exploring the potential role of GLP-1 signaling in perioperative renal physiology.

While the precise mechanisms remain to be clarified, elevated GLP-1 levels in postoperative AKI may reflect impaired renal clearance, metabolic stress responses, or early counter-regulatory activation. From a translational perspective, baseline GLP-1 assessment before surgery in patients without manifest renal dysfunction may contribute to improved understanding of perioperative renal vulnerability. While serum creatinine remains the clinical standard for AKI diagnosis, it is a delayed marker, whereas early biomarkers such as IL-18 and NGAL were not assessed in this study; moreover, the predefined sampling time points did not capture the early postoperative phase relevant for some of these markers. Therefore, the incremental predictive value of GLP-1 beyond established biomarkers cannot be determined. Future investigations should aim to validate these findings in larger multicenter cohorts, refine threshold values, and evaluate GLP-1 within multimarker panels including established predictors such as interleukin-18, NGAL, and suPAR, thereby enhancing perioperative risk stratification^13^. Given the observational design of the present study, the reported associations do not imply causality but rather highlight GLP-1 as a promising candidate for further mechanistic investigation.

### Study limitations

This single-centre observational study included a relatively small sample size (*n* = 107), limiting generalizability. This analysis was not prespecified in the parent study protocol and should therefore be considered exploratory. Furthermore, the study population represents a subset of the larger ongoing biobank cohort (ClinicalTrials.gov identifier: NCT02488876), selected based on the availability of complete biomarker and outcome data at the time of analysis, which may introduce selection bias. The number of AKI events (*n* = 21) limited the scope of multivariable modelling; thus, regression analyses were performed in an exploratory manner and non‑parametric comparisons were used to minimise overfitting. In addition, multivariable regression and time-to-event analyses were performed in an exploratory manner due to the limited number of events and should be interpreted with caution. AKI was defined solely by KDIGO creatinine criteria without urinary biomarkers, histology, or long‑term renal outcomes. Due to the observational design, causal inference is not possible. Although GLP-1 demonstrated numerically higher AUC values than creatinine, AUC values remained moderate (< 0.70), likely reflecting sample size and clinical heterogeneity. The wide and overlapping confidence intervals further indicate that discrimination remains modest and that the differences should be interpreted cautiously. Whether combining GLP‑1 with other biomarkers (e.g. suPAR, NGAL, IL‑18) could further characterise its association with AKI warrants future evaluation. As only elective cardiac surgery patients were included, findings may not generalise to emergency or non‑cardiac settings. In addition, potential confounding by differences in surgical procedure type cannot be fully excluded. Finally, GLP‑1 and GIP were measured using a single assay platform; although performed in duplicate, inter‑assay differences across platforms cannot be excluded. Accordingly, the exploratory design and moderate discrimination preclude immediate clinical application and underscore the need for further evaluation in independent cohorts.

## Conclusions

This prospective study demonstrates an association between perioperative GLP-1 levels and postoperative AKI after cardiac surgery. Elevated preoperative GLP-1 levels were significantly associated with postoperative AKI and were elevated earlier in the perioperative course compared with serum creatinine, particularly in patients without pre-existing CKD. In contrast, GIP showed no association with AKI at any perioperative time point, while creatinine remained a delayed indicator of established renal dysfunction.

These findings suggest that GLP-1 may reflect early kidney stress or subclinical vulnerability preceding overt renal injury, potentially linked to systemic inflammation, metabolic stress, or microcirculatory dysregulation. Whether perioperative GLP-1 assessment has implications for clinical risk evaluation or management remains to be determined.

Future studies should validate these findings in larger, multicenter cohorts, define clinically meaningful biomarker thresholds, and assess the additive value of GLP-1 within multimarker approaches. Such approaches may help to further clarify the biological and clinical relevance of GLP-1 in perioperative renal injury.

## Supplementary Information

Below is the link to the electronic supplementary material.

Supplementary Material 1

## Data Availability

The data sets generated and analyzed during the current study are not publicly available due to privacy and ethical restrictions but are available from the corresponding author on reasonable request.
