# Supplementary material for: Glucagon-like peptide-1 (GLP-1) levels are associated with acute kidney injury after cardiac surgery
Source: Sci Rep. 2026 Apr 25;16:13408. doi: 10.1038/s41598-026-48483-6 (PMC13110359; doi:10.1038/s41598-026-48483-6)
Supplement: Supplementary file 1 — Supplementary Material 1 [file 41598_2026_48483_MOESM1_ESM.docx]

**Figure 4**

**Kaplan–Meier analysis of AKI-free survival stratified by preoperative GLP-1 levels.**

Kaplan–Meier curves for AKI-free survival in patients with preoperative GLP-1 levels above versus below the ROC-derived Youden cut-off (30.3 pmol/L). Patients with elevated GLP-1 levels showed a significantly lower AKI-free survival (log-rank p = 0.003). Univ. Cox regression: HR 3.95 (95% CI: 1.46, 10.72), p = 0.007

|  | **n** | **AKI events** | **HR (95% CI)** | **p** |
| --- | --- | --- | --- | --- |
| **Univariable** |  |  |  |  |
| **GLP-1*** | 106 | 22 | 3.95 (1.46, 10.72) | 0.007 |
|  |  |  |  |  |
| **Multivariable** |  |  |  |  |
| Model 1 | 106 | 22 | 4.14 (1.50, 11.44) | 0.006 |
| Model 2 | 105 | 22 | 3.97 (1.44, 10.95) | 0.008 |
| Model 3 | 103 | 22 | 3.86 (1.42, 10.46) | 0.008 |
| Model 4 | 106 | 22 | 4.24 (1.48, 12.16) | 0.007 |

Model 1: Adjusted for age and sex, Model 2: Adjusted for BMI and creatinine, Model 3: Adjusted for eGFR and leucocyte count, Model 4: Adjusted for pre-existing CKD and complexity of surgery, *Preoperative GLP-1 levels above the ROC-derived Youden cut-off (30.3 pmol/L).

Multivariable models were performed in an exploratory manner due to the limited number of events.
